# Astrocyte-Derived Paracrine Signals: Relevance for Neurogenic Niche Regulation and Blood–Brain Barrier Integrity

**DOI:** 10.3389/fphar.2019.01346

**Published:** 2019-11-21

**Authors:** Simona Federica Spampinato, Valeria Bortolotto, Pier Luigi Canonico, Maria Angela Sortino, Mariagrazia Grilli

**Affiliations:** ^1^Section of Pharmacology, Department of Biomedical and Biotechnological Sciences, University of Catania, Catania, Italy; ^2^Laboratory of Neuroplasticity, Department of Pharmaceutical Sciences, University of Piemonte Orientale, Novara, Italy

**Keywords:** astrocytes, blood–brain barrier, neural stem cells, neurogenesis, niche, paracrine signals, secretome

## Abstract

Astrocytes are essential for proper regulation of the central nervous system (CNS). Importantly, these cells are highly secretory in nature. Indeed they can release hundreds of molecules which play pivotal physiological roles in nervous tissues and whose abnormal regulation has been associated with several CNS disorders. In agreement with these findings, recent studies have provided exciting insights into the key contribution of astrocyte-derived signals in the pleiotropic functions of these cells in brain health and diseases. In the future, deeper analysis of the astrocyte secretome is likely to further increase our current knowledge on the full potential of these cells and their secreted molecules not only as active participants in pathophysiological events, but as pharmacological targets or even as therapeutics for neurological and psychiatric diseases. Herein we will highlight recent findings in our and other laboratories on selected molecules that are actively secreted by astrocytes and contribute in two distinct functions with pathophysiological relevance for the astroglial population: i) regulation of neural stem cells (NSCs) and their progeny within adult neurogenic niches; ii) modulation of the blood–brain barrier (BBB) integrity and function.

## Introduction

Astrocytes are essential for brain homeostasis. They indeed support neurons both structurally and functionally by providing nutrients and neurotrophic factors, removing neurotransmitters and waste metabolites to ensure a homeostatic environment (Perez-Alvarez and Araque, 2013). Astrocytes regulate neurogenesis, axonal guidance, synaptogenesis (Allen and Lyons, 2018), as well as blood–brain barrier (BBB) function. Although still controversial, astrocytes may also release gliotransmitters to modulate synaptic transmission (Araque et al., 2014; Fiacco and McCarthy, 2018). Last but not least, after brain injury, astrocytes are involved in neuroinflammatory responses in an attempt of repair and/or remodeling.

Astrocytes are highly secretory cells, with their secretome containing hundreds of molecules (Chen and Swanson, 2003; Dowell et al., 2009; Harada et al., 2015). Recent proteomic studies provided exciting insights into the contribution of astrocyte-derived signals in their pleiotropic functions in brain health and diseases (Jha et al., 2018). In this minireview, we will highlight recent findings on some molecules actively secreted by astrocytes and implicated in two specific functions, namely, regulation of neural stem cells (NSCs) and their progeny within adult neurogenic niches and modulation of BBB function. These apparently distant conditions are analyzed together as they share a strict dependence on astrocyte-secreted products.

## Astrocytes as Key Modulators in Adult Neurogenic Niches

The term adult neurogenesis (aNG) refers to the generation of new functionally integrated neurons in the adult brain. This peculiar form of neuroplasticity occurs in restricted areas of mammalian brain, the subventricular zone (SVZ) in the lateral ventricles and the subgranular zone (SGZ) in the hippocampal dentate gyrus (DG).

While the SVZ region is considered a poorly relevant neurogenic site in humans, neurogenesis occurring in the DG appears of physiological significance. Recently, the presence of thousands of adult-born neuroblasts (NBs) in the hippocampus of healthy people was described (Moreno-Jimenez et al., 2019). In this region, neural stem/progenitor cells (NSC/NPC) self-renew and give rise to transiently amplifying progenitor cells which, in turn, can generate NBs capable of terminal neuronal differentiation. Post-mitotic neuronal progeny can be functionally integrated as granule cells into the adult hippocampal circuitry (Bond et al., 2015; Kempermann et al., 2015). In recent years, adult hippocampal neurogenesis (ahNG) has attracted growing interest due to its potential involvement in cognition and memory, mood, and emotional behavior (Aimone et al., 2010; Eisch and Petrik, 2012; Aimone et al., 2014; Bortolotto et al., 2014). ahNG is profoundly dysregulated in several neuropsychiatric/neurodegenerative disorders opening to the possibility that it may participate in their pathophysiology or contribute to some associated symptoms, such as dementia and depressed mood (Grilli and Meneghini, 2012; Bortolotto and Grilli, 2016; Yun et al., 2016). Very recently, it has been reported that postmortem tissue from AD patients contained remarkably fewer DG NBs suggesting their degeneration in the disease (Moreno-Jimenez et al., 2019). This seminal paper confirmed previous key studies in the field (Spalding et al., 2013; Boldrini et al., 2018).

**Figure 1:**
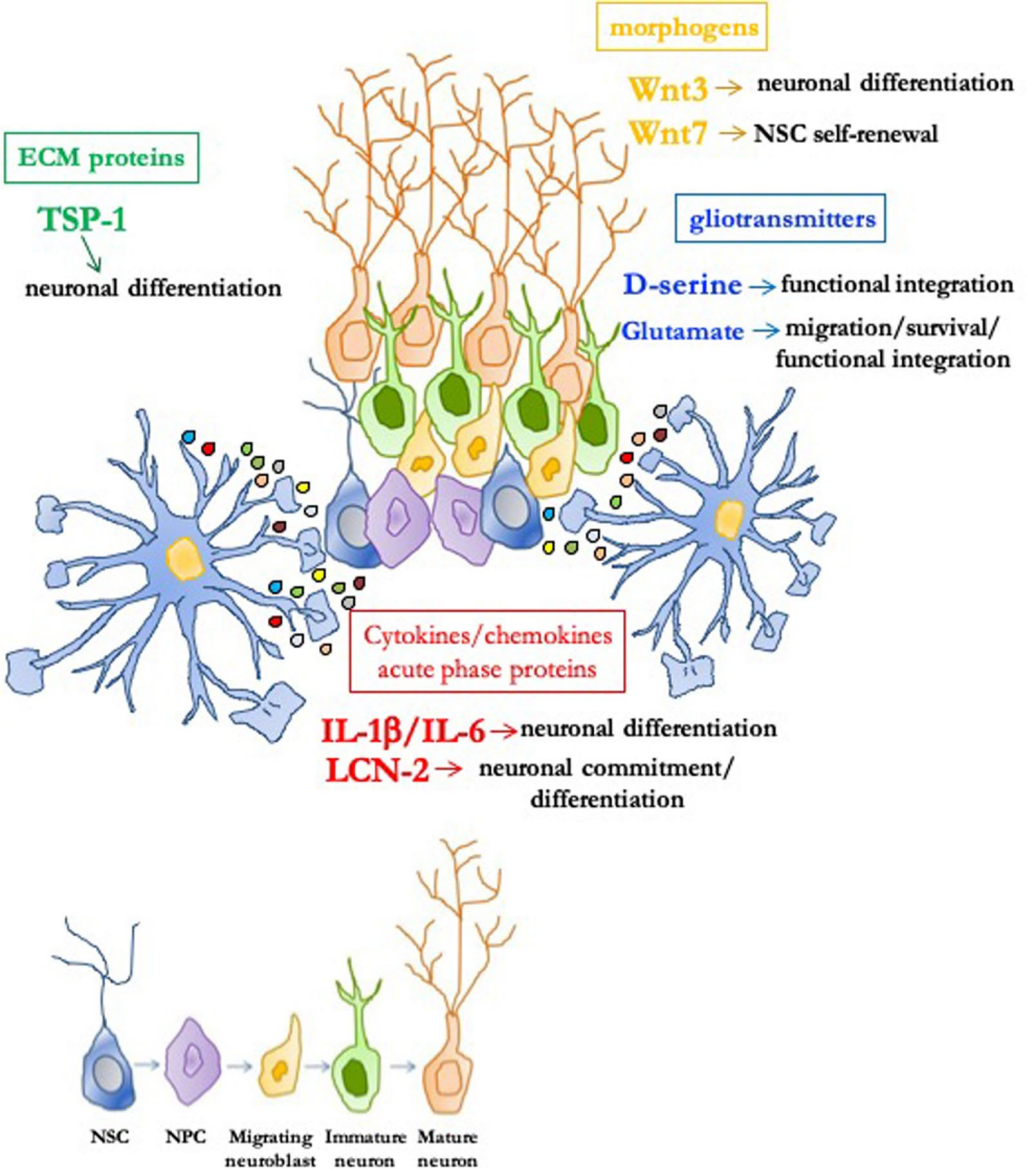
Role of astrocyte-derived molecules in the adult neurogenic niche. In the permissive and instructive microenvironment of the neurogenic niche, astrocytes profoundly modulate adult neurogenesis through soluble signals. Neural stem/progenitor cells (NSC/NPC) self-renewal, neuronal commitment/differentiation, migration of neuroblasts, as well as survival and functional integration of newly born neurons can be affected by different classes of astrocytic-derived factors such as morphogens (i.e., Wnt3 and Wnt7), gliotransmitters (i.e., D-serine and glutamate), extracellular matrix (ECM) proteins [i.e., thrombospondin 1 (TSP-1)], and cytokines/chemokines/acute phase proteins [i.e., IL-1β, IL-6, and lipocalin-2 (LCN-2)].

An important functional and anatomical concept in aNG is the "neurogenic niche," a permissive and instructive microenvironment which is crucial for preserving NSC functions, including their proliferative and differentiative properties (Ghosh, 2019). Although cell–cell contacts are relevant, paracrine signals originating from astrocytes within the niche appear very important. It was demonstrated that astrocytes are important neurogenic niche components which instruct NSC/NPC to adopt a neuronal fate (Song et al., 2002). Hence, the interest in the identity of astrocyte-secreted niche signals has been growing (Casse et al., 2018). We will now highlight key findings showing how astrocytes modulate aNG through release of different classes of secretory substances, as summarized in **Figure 1**.

### Morphogens

Among the first candidate molecules identified for their role in aNG were morphogenic factors of the Wnt protein family. Several members, including Wnt3 and Wnt7, are expressed by hippocampal astrocytes together with Wnt receptors and Wnt/β-catenin signaling pathway components (Lie et al., 2005). Hippocampal niche astrocytes actively induce ahNG through secretion of Wnt proteins and activation of Wnt downstream signaling pathways. Overexpression of Wnt3 enhances neuronal differentiation, while blockade of Wnt signaling strongly reduces ahNG *in vivo* and *in vitro* (Lie et al., 2005). Moreno-Estelles demonstrated that Wnt7a released by astrocytes in the adult neurogenic niche is a key factor promoting NSC self-renewal (Moreno-Estelles et al., 2012).

### Gliotransmitters

D-serine and Glutamate (Glu) were identified as molecules by which niche astrocytes regulate maturation, survival, and functional integration into local synaptic networks of adult-born neurons. To investigate the role of astrocytic exocytosis on aNG, SNAP Receptor protein (SNARE)-dependent exocytosis was genetically impaired in niche astrocytes (Sultan et al., 2015). Inhibition of vesicular release resulted in severely impaired integration and survival of newly generated hippocampal neurons, whereas developmentally born neurons appeared unaffected (Sultan et al., 2015). Adult-born neurons located within the territories of exocytosis-deficient astrocytes displayed reduced dendritic spine density and glutamatergic synaptic input, which correlated with decreased D-serine. Chronic administration of D-serine partially rescued defective phenotypes and restored N-methyl-D-aspartate (NMDA)-mediated synaptic transmission and dendritic maturation in mice with impaired astrocytic vesicular release (Sultan et al., 2015). The observation that rescue was only partial suggested that other molecules released by astrocytes could be important for maturation of adult-born hippocampal neurons. A critical role of vesicular Glu release from astrocytes was previously demonstrated in the SVZ where newly generated NBs migrate a long distance to reach their final destination, the olfactory bulb (OB). Platel et al. demonstrated that migrating NBs, which acquire functional NMDA receptor activity on their way to the OB, are ensheathed by astrocytes releasing glutamate in a Ca^2+^-dependent manner (Platel et al., 2010). They showed that: i) increasing calcium in astrocytes induced NMDA receptor activity in NB; ii) blocking vesicular astrocytic release eliminated spontaneous NMDA receptor activity in NB; and iii) deletion of functional NMDA receptors in adult-born NB decreased generation and survival of newborn neurons in adult OB (Platel et al., 2010). Altogether, these findings correlate astrocyte-released Glu with generation, survival, and functional integration into local synaptic networks of adult-born OB neurons.

### Extracellular Matrix (ECM) Proteins

Several astrocyte-secreted ECM proteins modulate cellular functions. Usually these proteins are expressed at high levels during development and at lower levels in adulthood. Upon brain injury, their expression is upregulated, especially in reactive astrocytes, and they are often associated with CNS remodeling and synaptogenesis. Some ECM proteins play also an important role in the neurogenic microenvironment. The most investigated astrocyte-secreted matricellular proteins are thrombospondins (TSPs) which mediate cell–cell and cell–matrix interaction by binding other ECM components, membrane receptors, growth factors, and cytokines. TSP-1 represents a key astrocyte-derived pro-neurogenic factor which promotes neuronal differentiation of NSC (Lu and Kipnis, 2010). Adult TSP-1^−/−^ mice exhibit reduced NSC proliferation and neuronal differentiation in both SVZ and SGZ (Lu and Kipnis, 2010). The voltage-gated calcium channel α2δ1 subunit was proposed to be a receptor which mediates TSP-1 synaptogenic effects (Eroglu et al., 2009). The α2δ1 subunit was also proven to be functionally expressed by adult hippocampal NPC and to mediate TSP-1 and pregabalin (an anticonvulsant/analgesic α2δ1 ligand) pro-neurogenic effects both *in vitro* and *in vivo* (Valente et al., 2012). These findings were further extended in recent studies proposing a key role for nuclear factor kappa-light-chain-enhancer of activated B cells (NF-κB) signaling whose activation occurs in adult NSC *via* membrane receptors, including neurotransmitter receptors and α2δ1 (Meneghini et al., 2010; Bortolotto et al., 2017a; Bortolotto et al., 2019). NF-κB p50^−/−^ mice exhibit strongly reduced ahNG *in vivo* (Denis-Donini et al., 2008) and *in vitro* (Meneghini et al., 2013; Valente et al., 2015; Bonini et al., 2016). Interestingly, TSP-1 promotes an increase in the percentage of newly formed neurons in wild type, but not in p50^−/−^-derived ahNPC which have reduced α2δ1 expression levels (Cvijetic et al., 2017). Altogether, these data suggested that a disturbed astrocyte–NSC communication *via* TSP-1 may contribute to defects in ahNG in absence of p50.

### Cytokines and Acute Phase Proteins

In contrast with the notion that inflammatory cytokines inhibit neuronal differentiation (Vallieres et al., 2002; Monje et al., 2003), IL-1β and IL-6, both highly expressed in neurogenic niches astrocytes, strongly promote NSC neuronal differentiation (Barkho et al., 2006). Lipocalin-2 (LCN-2) is an acute phase protein produced by and acting on astrocytes (Jha et al., 2015) which serves as "help-me" signal to activate astrocytes and microglia (Xing et al., 2014). Although its modulatory role in the CNS is not completely understood LCN-2 is commonly regarded as a deleterious signal (Ferreira et al., 2015) and a key target in regulating astrocyte reactivity. Indeed it has been demonstrated that knockdown of LCN-2 leads to reduced protein secretion from reactive astroglial cells, counteracting the perpetuation of inflammation in nearby astrocytes (Smith et al., 2018). LCN-2 is encoded by a NF-κB target gene (Uberti et al., 2000), and its expression is increased in the secretome of p50^−/−^ astrocytes (Cvijetic et al., 2017; Bortolotto and Grilli, 2017b). Initially, based on these findings and its deleterious effects, our group hypothesized that overexpressed LCN-2 may contribute to impaired ahNG in p50^−/−^ mice. To our surprise, LCN-2 promoted, in a concentration-dependent manner, neuronal differentiation of ahNPC. Under the same experimental conditions, LCN-2 had little effect on neuronal differentiation of p50^−/−^ ahNPC which exhibited downregulation of the LCN-2 receptor 24p3R (Cvijetic et al., 2017). Altogether, these novel data proposed LCN-2 as a novel and unexpected astroglial-derived signal able to promote neuronal fate specification of ahNPC (Bortolotto and Grilli, 2017b). Recently, these findings were further extended by the demonstration that LCN-2^−/−^ mice display deficits in proliferation and neuronal commitment of NSC and, in parallel, hippocampal dysfunction (Ferreira et al., 2018).

In summary, at present several astrocyte-derived signals which act as positive modulators of NSC and their progeny have been identified and characterized. Of note, little is currently known on soluble molecules of astrocytic origin which may exert negative effects on aNG. Anatomical and functional segregation along the hippocampal dorso-ventral axis is a well-established concept (Grilli et al., 1988; Tanti and Belzung, 2013), and marked differences in neurogenesis rate have been described in the dorsal compared to the ventral dentate gyrus (Piatti et al., 2011). It would be interesting to investigate whether subregional specificities in ahNG may also rely, at least in part, on different astrocyte-secreted molecules.

## The Dual Role of Astrocytic-Derived Factors: From Endothelial Protection to Disruption of Bbb Function

The BBB is constituted by specialized endothelial cells, supported in their functions by astrocytes and pericytes, and is part of a more complex network, the neurovascular unit (NVU), that includes also microglia, neurons, and mast cells. Brain microvascular endothelial cells, the main anatomical BBB elements, express tight junctions (TJs) and adherens junctions (AJs) (Huber et al., 2001; Dejana and Giampietro, 2012), that allow a selective para- and transcellular movement of molecules and solutes across the barrier (Garg et al., 2008; Garcia et al., 2014). Trafficking through the BBB is regulated by specific transporters (Kastin and Pan, 2008), as well as by efflux pumps such as P-glycoprotein (P-gp) (Begley, 2004). The BBB contributes to make CNS a site of immune privilege, as low expression of adhesion molecules and tightness of cell-to-cell connections limit the access of pathogens and immune cells, preserving immune surveillance (Engelhardt and Ransohoff, 2005).

Astrocytes appear fundamental in BBB function. *In vitro*, barrier properties are lost in the absence of astrocytes (Ghazanfari and Stewart, 2001) and reestablished by astrocyte conditioned media or when astrocyte–endothelial cells contact is provided (Tao-Cheng et al., 1987; Neuhaus et al., 1991; Hayashi et al., 1997; Colgan et al., 2008). Further, endothelial cells derived from non-CNS districts, cultured in the presence of astrocytes or astrocyte-secreted factors, acquire BBB specific features, including expression of TJ or P-gp (Prat et al., 2001; Abbott et al., 2006).

Pericytes and radial glia, the major source of astrocyte precursors (McDermott et al., 2005), are essential in an early stage of barrier induction, whereas astrocytes play a major role later on, favoring barrier maturation and maintenance (Obermeier et al., 2013; Obermeier et al., 2016).

**Figure 2:**
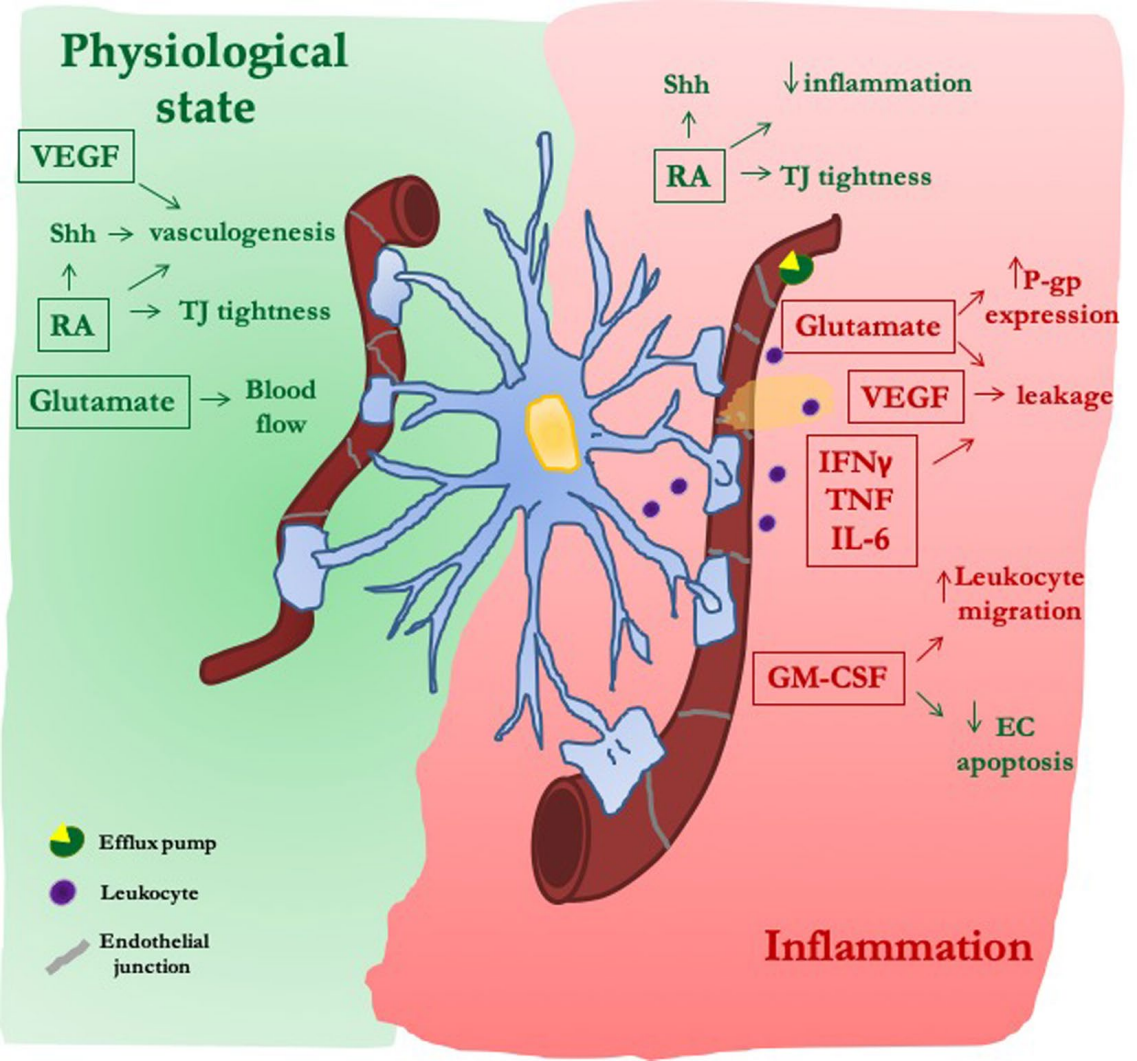
The dual role of astrocytic-derived factors on blood–brain barrier (BBB). Under physiological conditions, astrocytes release morphogens [sonic hedgehog (Shh) and retinoic acid (RA)], trophic factors (VEGF), and gliotransmitters (Glu) that, reinforcing both the formation of new vessels and the tightness of their junctions, improve the proper endothelial function at the BBB. After inflammatory stimuli, secretion of morphogens (Shh and RA) is reactivated in an attempt to reduce the inflammatory-mediated damage on endothelial layer. On the contrary, VEGF and Glu induce junctional damage and BBB leakiness, as well as increased expression of efflux pumps [P-glycoprotein (P-gp)]. The secretion of cytokines and chemokines is further increased, thus facilitating BBB leakage and leukocyte migration.

In pathological conditions, morphological changes in reactive astrocytes may induce loss of their interaction with endothelial cells (Alvarez et al., 2013). Depending on insult type, astrocytes undergo loss-of-function [e.g., failure of glutamate uptake (Broux et al., 2015)] and/or gain-of-function [production of a wide range of molecules including cytokines (Gimsa et al., 2013; Brambilla, 2019)]. All these events can lead to reduction or exacerbation of BBB damage. Herein we will analyze the crosstalk between astrocytes and endothelial cells in BBB function, focusing on few astrocytic soluble mediators that belong to the classes discussed above (**Figure 2**).

### Morphogens

Sonic hedgehog (Shh) is one of the main mediators of BBB induction. It is expressed in astrocytes, and its receptor has been detected *in vivo* in mice and human blood vessels as well as in cultured BBB endothelial cells (Alvarez et al., 2011). Its genetic deletion results in reduced expression of endothelial junctional proteins and accumulation of solutes in CNS (Alvarez et al., 2011). Shh is overexpressed in astrocytes following an ischemic insult and reinforces junctional tightness (Liu et al., 2019) thus reducing BBB leakage and brain edema (Xia et al., 2013). Accordingly, Shh mimetics promote immune-quiescence dampening leukocyte extravasation into the CNS, through the downregulation of adhesion molecules, as observed both *in vitro* (Alvarez et al., 2011) and *in vivo* (Singh et al., 2017). Retinoic acid (RA) is produced by radial glia and behaves as a morphogen playing a main role during brain development (Halilagic et al., 2007; Mizee et al., 2013). It is enhanced in reactive astrocytes after middle cerebral artery occlusion (Kong et al., 2015) and contributes to ameliorate barrier properties. RA indeed reinforces the expression of junctional proteins and P-gp in endothelial cells (Mizee et al., 2013) and reduces inflammatory genes (IL-6, CCL2, and VCAM-1) (Mizee et al., 2014). It also modifies ICAM-1 glycan composition (Chen et al., 2012), affecting the interaction of endothelial cells with PBMCs, an event that is modulated by astrocytes (Spampinato et al., 2019). Thus, beyond their physiological function in barrierogenesis, both Shh and RA play a role in the delay of BBB breakdown under pathological conditions.

### Trophic Factors

The main vascular trophic factor is VEGF-A. In contrast to its main activity in promoting angiogenesis, proliferation, differentiation, and survival of endothelial cells during brain development (Esser et al., 1998; Zhao et al., 2015), in adulthood VEGF is a potent inducer of BBB disruption. Reactive astrocytes are VEGF-A primary source and increased BBB immunoreactivity is often observed in animal models of multiple sclerosis (Maharaj and D'Amore, 2007; Argaw et al., 2012), Alzheimer's disease (Zand et al., 2005), ischemia, and traumatic brain injury (Shore et al., 2004; Jiang et al., 2014; Wu et al., 2018). Acting either directly on its receptors on endothelial cells, (Argaw et al., 2012; Chapouly et al., 2015), or indirectly, through the modulation of matrix metalloproteinases (MMPs) (Michinaga et al., 2015; Spampinato et al., 2017), VEGF-A induces changes in the tightness of endothelial junctions, causing brain edema, as well as leukocyte adhesion and infiltration in the CNS. Accordingly, blockade of VEGF-A through specific antibodies alleviates BBB disruption (Michinaga et al., 2018), whereas VEGF-A knockdown in astrocytes results in reduced endothelial expression of MMP9 and prevention of barrier leakage (Spampinato et al., 2017).

### Cytokines and Chemokines

Cytokines released by reactive astrocytes in close proximity to the BBB induce TJ re-organization [TNF and IFNγ (Chaitanya et al., 2011), CCL2 (Yao and Tsirka, 2014)], and immune cells recruitment [CXCL10, CCL2, CCL5, IL-8, CXCL12 (Brambilla, 2019)], further contributing to neuroinflammation. By stimulating proteosomal degradation of junctional proteins (Chang et al., 2015), astrocyte-derived IL-6 increases barrier permeability and the release of chemokines, thus enhancing PBMCs' access into the CNS (Takeshita et al., 2017). Astrocytes may also mediate endothelial responses to cytokines. Their presence is in fact necessary for INFγ to affect barrier properties, whereas only slight effects are reported on endothelial cells cultured alone (Chaitanya et al., 2011). Conversely, astrocytes counteracted increased barrier permeability induced by TNF alone, or in association with IL-6, on induced plutipotent stem cells-derived endothelial cells. The modulation of BBB properties by astrocyte-derived factors appears to be the result of a complex balance. Indeed, stressed astrocytes release not only factors triggering barrier breakdown (i.e., IFNγ, IL-1β, CCL5, CCL2, and CCL4), but anti-inflammatory ones like IL-4 (Mantle and Lee, 2018).

In addition, among astrocyte-derived factors, granulocyte and macrophage colony-stimulating factor (GM-CSF) exhibits a dual and controversial role. While promoting TJ internalization and downregulation (Shang et al., 2016; Zhang et al., 2018) and monocyte migration through the BBB (Vogel et al., 2015), GM-CSF also protects endothelial cells from apoptosis (Spampinato et al., 2015), induces claudin-5 overexpression (Shang et al., 2016), and stimulates angiopoietin-1 release from pericytes, thereby reducing barrier permeability (Yan et al., 2017).

### Gliotransmitters

Glu modifies BBB function through interaction with endothelial NMDA and metabotropic glutamate receptors. Activated astrocytes release large amounts of glutamate that act on endothelial NMDA receptors and promote oxidative stress (Scott et al., 2007), TJ disruption, and increased BBB permeability (Andras et al., 2007). Further, glutamate increases the expression of P-gp, as reported in endothelial cells cultured with astrocytes derived from amyotrophic lateral sclerosis (ALS) patients (Mohamed et al., 2019). This condition can justify "P-gp–mediated pharmacoresistance" (Mohamed et al., 2017), often observed in diseases including ALS and epilepsy (Avemary et al., 2013; Feldmann et al., 2013).

## Concluding Remarks

Our current understanding of the role of astrocytes in adult mammalian brain is growing exponentially, unraveling a remarkable variety of functions under the control of these cells both under physiological and pathological conditions. In recent years, the fact that astrocytes execute many of their crucial functions in a paracrine manner is also providing fuel to major advancements in astrocyte biology. Several proteins identified in studies that have applied proteomics for comprehensive profiling of astrocyte-secreted proteins confirmed that many of them correlate with well-known astrocyte-mediated cell-to-cell communication pathways. In some cases, soluble signals released by astrocytes *in vitro* created the opportunity to propose novel unexpected roles for these molecules and astrocytes. Hopefully, in the future, deeper analysis of the astrocyte secretome may further increase our current knowledge on the full potential of these cells and their secreted molecules not only as active participants in pathophysiological events, but as pharmacological targets or even as therapeutics for CNS diseases.

## Author Contributions

All authors contributed to the discussion, preparation, and proofreading of the manuscript.

## Funding

This work was partially supported by PRIN MIUR 2017. VB was supported by a SIF/MSD fellowship 2016. SFS was supported by a SIF/MSD fellowship 2017.

## Conflict of Interest

The authors declare that the research was conducted in the absence of any commercial or financial relationships that could be construed as a potential conflict of interest.

## Abbreviations

ahNG, adult hippocampal neurogenesis; AJ, adherens junctions; aNG, adult neurogenesis; CCL, CC Chemokine Ligand; CXCL, C-X-C motif chemokine 12; DG, dentate gyrus; ECM, extracellular matrix; ICAM-1, intercellular adhesion molecule 1; IFNγ, interferon γ; IL-1, interleukin 1; IL-6, interleukin 6; LCN-2, lipocalin-2; NB, neuroblast; NPC, neural progenitor cell; NSC, neural stem cell; NVU, neurovascular unit; OB, olfactory bulb; PBMCs, peripheral blood mononuclear cells; SGZ, subgranular zone; SVZ, subventricular zone; TNFα, tumor necrosis factor α; TSP, thrombospondin; VCAM, vascular cell adhesion molecule; VEGF, vascular endothelial growth factor.

## References

[B1] Abbott N. J. Ronnback L. Hansson E. (2006). Astrocyte-endothelial interactions at the blood-brain barrier. Nat. Rev. Neurosci. 7 (1), 41–53. 10.1038/nrn1824 16371949

[B2] Aimone J. B. Deng W. Gage F. H. (2010). Adult neurogenesis: integrating theories and separating functions. Trends Cognit. Sci. 14 (7), 325–337. 10.1016/j.tics.2010.04.003 20471301 PMC2904863

[B3] Aimone J. B. Li Y. Lee S. W. Clemenson G. D. Deng W. Gage F. H. (2014). Regulation and function of adult neurogenesis: from genes to cognition. Physiol. Rev. 94 (4), 991–1026. 10.1152/physrev.00004.2014 25287858 PMC4280160

[B4] Allen N. J. Lyons D. A. (2018). Glia as architects of central nervous system formation and function. Sci. 362 (6411), 181–185. 10.1126/science.aat0473 PMC6292669 30309945

[B5] Alvarez J. I. Dodelet-Devillers A. Kebir H. Ifergan I. Fabre P. J. Terouz S. (2011). The hedgehog pathway promotes blood-brain barrier integrity and CNS immune quiescence. Sci. 334 (6063), 1727–1731. 10.1126/science.1206936 22144466

[B6] Alvarez J. I. Katayama T. Prat A. (2013). Glial influence on the blood brain barrier. Glia 61 (12), 1939–1958. 10.1002/glia.22575 24123158 PMC4068281

[B7] Andras I. E. Deli M. A. Veszelka S. Hayashi K. Hennig B. Toborek M. (2007). The NMDA and AMPA/KA receptors are involved in glutamate-induced alterations of occludin expression and phosphorylation in brain endothelial cells. J. Cereb. Blood Flow Metab. 27 (8), 1431–1443. 10.1038/sj.jcbfm.9600445 17245419

[B8] Araque A. Carmignoto G. Haydon P. G. Oliet S. H. Robitaille R. Volterra A. (2014). Gliotransmitters travel in time and space. Neuron 81 (4), 728–739. 10.1016/j.neuron.2014.02.007 24559669 PMC4107238

[B9] Argaw A. T. Asp L. Zhang J. Navrazhina K. Pham T. Mariani J. N. (2012). Astrocyte-derived VEGF-A drives blood-brain barrier disruption in CNS inflammatory disease. J. Clin. Invest. 122 (7), 2454–2468. 10.1172/JCI60842 22653056 PMC3386814

[B10] Avemary J. Salvamoser J. D. Peraud A. Remi J. Noachtar S. Fricker G. (2013). Dynamic regulation of P-glycoprotein in human brain capillaries. Mol. Pharm. 10 (9), 3333–3341. 10.1021/mp4001102 23924183

[B11] Barkho B. Z. Song H. Aimone J. B. Smrt R. D. Kuwabara T. Nakashima K. (2006). Identification of astrocyte-expressed factors that modulate neural stem/progenitor cell differentiation. Stem Cells Dev. 15 (3), 407–421. 10.1089/scd.2006.15.407 16846377 PMC2777811

[B12] Begley D. J. (2004). ABC transporters and the blood-brain barrier. Curr. Pharm. Des. 10 (12), 1295–1312. 15134482 10.2174/1381612043384844

[B13] Boldrini M. Fulmore C. A. Tartt A. N. Simeon L. R. Pavlova I. Poposka V. (2018). Human hippocampal neurogenesis persists throughout aging. Cell Stem Cell 22 (4), 589–599 e585. 10.1016/j.stem.2018.03.015 29625071 PMC5957089

[B14] Bond A. M. Ming G. L. Song H. (2015). Adult mammalian neural stem cells and neurogenesis: five decades later. Cell Stem Cell 17 (4), 385–395. 10.1016/j.stem.2015.09.003 26431181 PMC4683085

[B15] Bonini S. A. Mastinu A. Maccarinelli G. Mitola S. Premoli M. La Rosa L. R. (2016). Cortical structure alterations and social behavior impairment in p50-deficient mice. Cereb Cortex 26 (6), 2832–2849. 10.1093/cercor/bhw037 26946128 PMC4869818

[B16] Bortolotto V. Bondi H. Cuccurazzu B. Rinaldi M. Canonico P. L. Grilli M. (2019). Salmeterol, a β2 adrenergic agonist, promotes adult hippocampal neurogenesis in a region-specific manner. Front. Pharmacol. 10, 1000. 10.3389/fphar.2019.01000 31572182 PMC6751403

[B17] Bortolotto V. Cuccurazzu B. Canonico P. L. Grilli M. (2014). NF-kappaB mediated regulation of adult hippocampal neurogenesis: relevance to mood disorders and antidepressant activity. BioMed. Res. Int. 2014, 612798. 10.1155/2014/612798 24678511 PMC3942292

[B18] Bortolotto V. Grilli M. (2016). Not only a bad guy: potential proneurogenic role of the RAGE/NF-kappaB axis in Alzheimer's disease brain. Neural Regener. Res. 11 (12), 1924–1925. 10.4103/1673-5374.197130 PMC5270427 28197185

[B19] Bortolotto V. Grilli M. (2017b). Novel insights into the role of NF-κB p50 in astrocyte - mediated fate specification of adult neural progenitor cells. Neural Regener. Res. 12 (3), 354–357. 10.4103/1673-5374.202919 PMC5399701 28469638

[B20] Bortolotto V. Mancini F. Mangano G. Salem R. Xia E. Del Grosso E. (2017a). Proneurogenic effects of trazodone in murine and human neural progenitor cells. ACS Chem. Neurosci. 8 (9), 2027–2038. 10.1021/acschemneuro.7b00175 28636360

[B21] Brambilla R. (2019). The contribution of astrocytes to the neuroinflammatory response in multiple sclerosis and experimental autoimmune encephalomyelitis. Acta Neuropathol. 137 (5), 757–783. 10.1007/s00401-019-01980-7 30847559 PMC6483860

[B22] Broux B. Gowing E. Prat A. (2015). Glial regulation of the blood-brain barrier in health and disease. Semin. Immunopathol. 37 (6), 577–590. 10.1007/s00281-015-0516-2 26245144

[B23] Casse F. Richetin K. Toni N. (2018). Astrocytes' contribution to adult neurogenesis in physiology and Alzheimer's disease. Front. Cell Neurosci. 12, 432. 10.3389/fncel.2018.00432 30538622 PMC6277517

[B24] Chaitanya G. V. Cromer W. E. Wells S. R. Jennings M. H. Couraud P. O. Romero I. A. (2011). Gliovascular and cytokine interactions modulate brain endothelial barrier in vitro. J. Neuroinflammation 8, 162. 10.1186/1742-2094-8-162 22112345 PMC3248576

[B25] Chang C. Y. Li J. R. Chen W. Y. Ou Y. C. Lai C. Y. Hu Y. H. (2015). Disruption of in vitro endothelial barrier integrity by Japanese encephalitis virus-Infected astrocytes. Glia 63 (11), 1915–1932. 10.1002/glia.22857 25959931

[B26] Chapouly C. Tadesse Argaw A. Horng S. Castro K. Zhang J. Asp L. (2015). Astrocytic TYMP and VEGFA drive blood-brain barrier opening in inflammatory central nervous system lesions. Brain 138 (Pt 6), 1548–1567. 10.1093/brain/awv077 25805644 PMC4614128

[B27] Chen C. Diao D. Guo L. Shi M. Gao J. Hu M. (2012). All-trans-retinoic acid modulates ICAM-1 N-glycan composition by influencing GnT-III levels and inhibits cell adhesion and trans-endothelial migration. PloS One 7 (12), e52975. 10.1371/journal.pone.0052975 23300837 PMC3530489

[B28] Chen Y. Swanson R. A. (2003). Astrocytes and brain injury. J. Cereb. Blood Flow Metab. 23 (2), 137–149. 10.1097/01.WCB.0000044631.80210.3C 12571445

[B29] Colgan O. C. Collins N. T. Ferguson G. Murphy R. P. Birney Y. A. Cahill P. A. (2008). Influence of basolateral condition on the regulation of brain microvascular endothelial tight junction properties and barrier function. Brain Res. 1193, 84–92. 10.1016/j.brainres.2007.11.072 18177846

[B30] Cvijetic S. Bortolotto V. Manfredi M. Ranzato E. Marengo E. Salem R. (2017). Cell autonomous and noncell-autonomous role of NF-kappaB p50 in astrocyte-mediated fate specification of adult neural progenitor cells. Glia 65 (1), 169–181. 10.1002/glia.23085 27758000

[B31] Dejana E. Giampietro C. (2012). Vascular endothelial-cadherin and vascular stability. Curr. Opin. Hematol. 19 (3), 218–223. 10.1097/MOH.0b013e3283523e1c 22395663

[B32] Denis-Donini S. Dellarole A. Crociara P. Francese M. T. Bortolotto V. Quadrato G. (2008). Impaired adult neurogenesis associated with short-term memory defects in NF-kappaB p50-deficient mice. J. Neurosci. 28 (15), 3911–3919. 10.1523/JNEUROSCI.0148-08.2008 18400889 PMC6670458

[B33] Dowell J. A. Johnson J. A. Li L. (2009). Identification of astrocyte secreted proteins with a combination of shotgun proteomics and bioinformatics. J. Proteome Res. 8 (8), 4135–4143. 10.1021/pr900248y 19469553 PMC2866504

[B34] Eisch A. J. Petrik D. (2012). Depression and hippocampal neurogenesis: a road to remission? Sci. 338 (6103), 72–75. 10.1126/science.1222941 PMC3756889 23042885

[B35] Engelhardt B. Ransohoff R. M. (2005). The ins and outs of T-lymphocyte trafficking to the CNS: anatomical sites and molecular mechanisms. Trends Immunol. 26 (9), 485–495. 10.1016/j.it.2005.07.004 16039904

[B36] Eroglu C. Allen N. J. Susman M. W. O'Rourke N. A. Park C. Y. Ozkan E. (2009). Gabapentin receptor alpha2delta-1 is a neuronal thrombospondin receptor responsible for excitatory CNS synaptogenesis. Cell 139 (2), 380–392. 10.1016/j.cell.2009.09.025 19818485 PMC2791798

[B37] Esser S. Wolburg K. Wolburg H. Breier G. Kurzchalia T. Risau W. (1998). Vascular endothelial growth factor induces endothelial fenestrations in vitro. J. Cell Biol. 140 (4), 947–959. 10.1083/jcb.140.4.947 9472045 PMC2141756

[B38] Feldmann M. Asselin M. C. Liu J. Wang S. McMahon A. Anton-Rodriguez J. (2013). P-glycoprotein expression and function in patients with temporal lobe epilepsy: a case-control study. Lancet Neurol. 12 (8), 777–785. 10.1016/S1474-4422(13)70109-1 23786896

[B39] Ferreira A. C. Da Mesquita S. Sousa J. C. Correia-Neves M. Sousa N. Palha J. A. (2015). From the periphery to the brain: Lipocalin-2, a friend or foe? Prog. Neurobiol. 131, 120–136. 10.1016/j.pneurobio.2015.06.005 26159707

[B40] Ferreira A. C. Santos T. Sampaio-Marques B. Novais A. Mesquita S. D. Ludovico P. (2018). Lipocalin-2 regulates adult neurogenesis and contextual discriminative behaviours. Mol. Psychiatry 23 (4), 1031–1039. 10.1038/mp.2017.95 28485407

[B41] Fiacco T. A. McCarthy K. D. (2018). Multiple lines of evidence indicate that gliotransmissiondoes not occur under physiological conditions. J. Neurosci. 38 (1), 3–13. 10.1523/JNEUROSCI.0016-17.2017 29298904 PMC5761435

[B42] Garcia K. O. Ornellas F. L. Martin P. K. Patti C. L. Mello L. E. Frussa-Filho R. (2014). Therapeutic effects of the transplantation of VEGF overexpressing bone marrow mesenchymal stem cells in the hippocampus of murine model of Alzheimer's disease. Front. Aging Neurosci. 6, 30. 10.3389/fnagi.2014.00030 24639647 PMC3945612

[B43] Garg S. K. Banerjee R. Kipnis J. (2008). Neuroprotective immunity: T cell-derived glutamate endows astrocytes with a neuroprotective phenotype. J. Immunol. 180 (6), 3866–3873. 10.4049/jimmunol.180.6.3866 18322194

[B44] Ghazanfari F. A. Stewart R. R. (2001). Characteristics of endothelial cells derived from the blood-brain barrier and of astrocytes in culture. Brain Res. 890 (1), 49–65. 10.1016/s0006-8993(00)03053-5 11164768

[B45] Ghosh H. S. (2019). Adult neurogenesis and the promise of adult neural stem cells. J. Exp. Neurosci. 13, 1179069519856876. 10.1177/1179069519856876 31285654 PMC6600486

[B46] Gimsa U. Mitchison N. A. Brunner-Weinzierl M. C. (2013). Immune privilege as an intrinsic CNS property: astrocytes protect the CNS against T-cell-mediated neuroinflammation. Mediators Inflammation 2013, 320519. 10.1155/2013/320519 PMC3760105 24023412

[B47] Grilli M. Meneghini V. (2012). “NF-κB proteins in adult neurogenesis: relevance for learning and memory in physiology and pathology,” in Transcription factors CREB and NF-κB: involvement in synaptic plasticity and memory formation. Ed. Bentham Science Publishers (Sharjah, United Arab Emirates), 79–96. 10.2174/978160805257811201010079

[B48] Grilli M. Nisoli E. Memo M. Missale C. Spano P. (1988). Pharmacological characterization of D1 and D2 dopamine receptors in rat limbocortical areas. II. Dorsal Hippocampus. Neurosci. Lett. 87, 253–258. 10.1016/0304-3940(88)90457-0 2898118

[B49] Halilagic A. Ribes V. Ghyselinck N. B. Zile M. H. Dolle P. Studer M. (2007). Retinoids control anterior and dorsal properties in the developing forebrain. Dev. Biol. 303 (1), 362–375. 10.1016/j.ydbio.2006.11.021 17184764

[B50] Harada K. Kamiya T. Tsuboi T. (2015). Gliotransmitter release from astrocytes: functional, developmental, and pathological implications in the brain. Front. Neurosci. 9, 499. 10.3389/fnins.2015.00499 26793048 PMC4709856

[B51] Hayashi Y. Nomura M. Yamagishi S. Harada S. Yamashita J. Yamamoto H. (1997). Induction of various blood-brain barrier properties in non-neural endothelial cells by close apposition to co-cultured astrocytes. Glia 19 (1), 13–26. 8989564

[B52] Huber J. D. Egleton R. D. Davis T. P. (2001). Molecular physiology and pathophysiology of tight junctions in the blood-brain barrier. Trends Neurosci. 24 (12), 719–725. 10.1016/s0166-2236(00)02004-x 11718877

[B53] Jha M. K. Kim J. H. Song G. J. Lee W. H. Lee I. K. Lee H. W. (2018). Functional dissection of astrocyte-secreted proteins: implications in brain health and diseases. Prog. Neurobiol. 162, 37–69. 10.1016/j.pneurobio.2017.12.003 29247683

[B54] Jha M. K. Lee S. Park D. H. Kook H. Park K. G. Lee I. K. (2015). Diverse functional roles of lipocalin-2 in the central nervous system. Neurosci. Biobehav. Rev. 49, 135–156. 10.1016/j.neubiorev.2014.12.006 25511817

[B55] Jiang S. Xia R. Jiang Y. Wang L. Gao F. (2014). Vascular endothelial growth factors enhance the permeability of the mouse blood-brain barrier. PloS One 9 (2), e86407. 10.1371/journal.pone.0086407 24551038 PMC3925082

[B56] Kastin A. J. Pan W. (2008). Blood-brain barrier and feeding: regulatory roles of saturable transport systems for ingestive peptides. Curr. Pharm. Des. 14 (16), 1615–1619. 10.2174/138161208784705423 18673203 PMC2750905

[B57] Kempermann G. Song H. Gage F. H. (2015). Neurogenesis in the Adult Hippocampus. Cold Spring Harb Perspect. Biol. 7 (9), a018812. 10.1101/cshperspect.a018812 26330519 PMC4563705

[B58] Kong L. Wang Y. Wang X. J. Wang X. T. Zhao Y. Wang L. M. (2015). Retinoic acid ameliorates blood-brain barrier disruption following ischemic stroke in rats. Pharmacol. Res. 99, 125–136. 10.1016/j.phrs.2015.05.014 26066585

[B59] Lie D. C. Colamarino S. A. Song H. J. Desire L. Mira H. Consiglio A. (2005). Wnt signalling regulates adult hippocampal neurogenesis. Nat. 437 (7063), 1370–1375. 10.1038/nature04108 16251967

[B60] Liu S. Chang L. Wei C. (2019). The sonic hedgehog pathway mediates Tongxinluo capsule-induced protection against blood-brain barrier disruption after ischaemic stroke in mice. Basic Clin. Pharmacol. Toxicol. 124 (6), 660–669. 10.1111/bcpt.13186 30548093

[B61] Lu Z. Kipnis J. (2010). Thrombospondin 1–a key astrocyte-derived neurogenic factor. FASEB J. 24 (6), 1925–1934. 10.1096/fj.09-150573 20124433 PMC3231793

[B62] Maharaj A. S. D'Amore P. A. (2007). Roles for VEGF in the adult. Microvasc Res. 74 (2–3), 100–113. 10.1016/j.mvr.2007.03.004 17532010 PMC2128714

[B63] Mantle J. L. Lee K. H. (2018). A differentiating neural stem cell-derived astrocytic population mitigates the inflammatory effects of TNF-alpha and IL-6 in an iPSC-based blood-brain barrier model. Neurobiol. Dis. 119, 113–120. 10.1016/j.nbd.2018.07.030 30075293

[B64] McDermott K. W. Barry D. S. McMahon S. S. (2005). Role of radial glia in cytogenesis, patterning and boundary formation in the developing spinal cord. J. Anat. 207 (3), 241–250. 10.1111/j.1469-7580.2005.00462.x 16185248 PMC1571535

[B65] Meneghini V. Bortolotto V. Francese M. T. Dellarole A. Carraro L. Terzieva S. (2013). High-mobility group box-1 protein and β-amyloid oligomers promote neuronal differentiation of adult hippocampal neural progenitors via receptor for advanced glycation end products/nuclear factor-κB axis: relevance for Alzheimer's disease. J. Neurosci. 33 (14), 6047–6059. 10.1523/JNEUROSCI.2052-12.2013 23554486 PMC6618915

[B66] Meneghini V. Francese M. T. Carraro L. Grilli M. (2010). A novel role for the receptor for advanced glycation end-products in neural progenitor cells derived from adult SubVentricular Zone. Mol. Cell Neurosci. 45 (2), 139–150. 10.1016/j.mcn.2010.06.005 20600932

[B67] Michinaga S. Kimura A. Hatanaka S. Minami S. Asano A. Ikushima Y. (2018). Delayed administration of BQ788, an ETB antagonist, after experimental traumatic brain injury promotes recovery of blood-brain barrier function and a reduction of cerebral edema in mice. J. Neurotrauma 35 (13), 1481–1494. 10.1089/neu.2017.5421 29316834

[B68] Michinaga S. Seno N. Fuka M. Yamamoto Y. Minami S. Kimura A. (2015). Improvement of cold injury-induced mouse brain edema by endothelin ETB antagonists is accompanied by decreases in matrixmetalloproteinase 9 and vascular endothelial growth factor-A. Eur. J. Neurosci. 42 (6), 2356–2370. 10.1111/ejn.13020 26174228

[B69] Mizee M. R. Nijland P. G. van der Pol S. M. Drexhage J. A. van Het Hof B. Mebius R. (2014). Astrocyte-derived retinoic acid: a novel regulator of blood-brain barrier function in multiple sclerosis. Acta Neuropathol. 128 (5), 691–703. 10.1007/s00401-014-1335-6 25149081

[B70] Mizee M. R. Wooldrik D. Lakeman K. A. van het Hof B. Drexhage J. A. Geerts D. (2013). Retinoic acid induces blood-brain barrier development. J. Neurosci. 33 (4), 1660–1671. 10.1523/JNEUROSCI.1338-12.2013 23345238 PMC6618717

[B71] Mohamed L. A. Markandaiah S. Bonanno S. Pasinelli P. Trotti D. (2017). Blood-brain barrier driven pharmacoresistance in amyotrophic lateral sclerosis and challenges for effective drug therapies. AAPS J. 19 (6), 1600–1614. 10.1208/s12248-017-0120-6 28779378 PMC6571115

[B72] Mohamed L. A. Markandaiah S. S. Bonanno S. Pasinelli P. Trotti D. (2019). Excess glutamate secreted from astrocytes drives upregulation of P-glycoprotein in endothelial cells in amyotrophic lateral sclerosis. Exp. Neurol. 316, 27–38. 10.1016/j.expneurol.2019.04.002 30974102 PMC6506236

[B73] Monje M. L. Toda H. Palmer T. D. (2003). Inflammatory blockade restores adult hippocampal neurogenesis. Sci. 302 (5651), 1760–1765. 10.1126/science.1088417 14615545

[B74] Moreno-Estelles M. Gonzalez-Gomez P. Hortiguela R. Diaz-Moreno M. San Emeterio J. Carvalho A. L. (2012). Symmetric expansion of neural stem cells from the adult olfactory bulb is driven by astrocytes via WNT7A. Stem Cells 30 (12), 2796–2809. 10.1002/stem.1243 22987443

[B75] Moreno-Jimenez E. P. Flor-Garcia M. Terreros-Roncal J. Rabano A. Cafini F. Pallas-Bazarra N. (2019). Adult hippocampal neurogenesis is abundant in neurologically healthy subjects and drops sharply in patients with Alzheimer's disease. Nat. Med. 25 (4), 554–560. 10.1038/s41591-019-0375-9 30911133

[B76] Neuhaus J. Risau W. Wolburg H. (1991). Induction of blood-brain barrier characteristics in bovine brain endothelial cells by rat astroglial cells in transfilter coculture. Ann. N Y Acad. Sci. 633, 578–580. 10.1111/j.1749-6632.1991.tb15667.x 1789585

[B77] Obermeier B. Daneman R. Ransohoff R. M. (2013). Development, maintenance and disruption of the blood-brain barrier. Nat. Med. 19 (12), 1584–1596. 10.1038/nm.3407 24309662 PMC4080800

[B78] Obermeier B. Verma A. Ransohoff R. M. (2016). The blood-brain barrier. Handb. Clin. Neurol. 133, 39–59. 10.1016/B978-0-444-63432-0.00003-7 27112670

[B79] Perez-Alvarez A. Araque A. (2013). Astrocyte-neuron interaction at tripartite synapses. Curr. Drug Targets 14 (11), 1220–1224. 10.2174/13894501113149990203 23621508

[B80] Piatti V. C. Davies-Sala M. G. Espósito M. S. Mongiat L. A. Trinchero M. F. Schinder A. F. (2011). The timing for neuronal maturation in the adult hippocampus is modulated by local network activity. J. Neurosci. 31 (21), 7715–7728. 10.1523/JNEUROSCI.1380-11.2011 21613484 PMC3701257

[B81] Platel J. C. Dave K. A. Gordon V. Lacar B. Rubio M. E. Bordey A. (2010). NMDA receptors activated by subventricular zone astrocytic glutamate are critical for neuroblast survival prior to entering a synaptic network. Neuron 65 (6), 859–872. 10.1016/j.neuron.2010.03.009 20346761 PMC2861893

[B82] Prat A. Biernacki K. Wosik K. Antel J. P. (2001). Glial cell influence on the human blood-brain barrier. Glia 36 (2), 145–155. 11596123 10.1002/glia.1104

[B83] Scott G. S. Bowman S. R. Smith T. Flower R. J. Bolton C. (2007). Glutamate-stimulated peroxynitrite production in a brain-derived endothelial cell line is dependent on N-methyl-D-aspartate (NMDA) receptor activation. Biochem. Pharmacol. 73 (2), 228–236. 10.1016/j.bcp.2006.09.021 17118345 PMC1855445

[B84] Shang S. Yang Y. M. Zhang H. Tian L. Jiang J. S. Dong Y. B. (2016). Intracerebral GM-CSF contributes to transendothelial monocyte migration in APP/PS1 Alzheimer's disease mice. J. Cereb. Blood Flow Metab. 36 (11), 1978–1991. 10.1177/0271678X16660983 27444968 PMC5094311

[B85] Shore P. M. Jackson E. K. Wisniewski S. R. Clark R. S. Adelson P. D. Kochanek P. M. (2004). Vascular endothelial growth factor is increased in cerebrospinal fluid after traumatic brain injury in infants and children. Neurosurgery 54 (3), 605–611. 10.1227/01.neu.0000108642.88724.db 15028134

[B86] Singh V. B. Singh M. V. Piekna-Przybylska D. Gorantla S. Poluektova L. Y. Maggirwar S. B. (2017). Sonic Hedgehog mimetic prevents leukocyte infiltration into the CNS during acute HIV infection. Sci. Rep. 7 (1), 9578. 10.1038/s41598-017-10241-0 28852071 PMC5575104

[B87] Smith J. A. Braga A. Verheyen J. Basilico S. Bandiera S. Alfaro-Cervello C. (2018). RNA nanotherapeutics for the amelioration of astroglial reactivity. Mol. Ther. Nucleic Acids 10, 103–121. 10.1016/j.omtn.2017.11.008 29499926 PMC5738063

[B88] Song H. Stevens C. F. Gage F. H. (2002). Astroglia induce neurogenesis from adult neural stem cells. Nat. 417 (6884), 39–44. 10.1038/417039a 11986659

[B89] Spalding K. L. Bergmann O. Alkass K. Bernard S. Salehpour M. Huttner H. B. (2013). Dynamics of hippocampal neurogenesis in adult humans. Cell 153 (6), 1219–1227. 10.1016/j.cell.2013.05.002 23746839 PMC4394608

[B90] Spampinato S. F. Merlo S. Fagone E. Fruciano M. Barbagallo C. Kanda T. (2019). Astrocytes modify migration of PBMCs induced by β-amyloid in a blood-brain barrier in vitro model. Front. In Cell. Neurosci. 13. 10.3389/fncel.2019.00337 PMC6664149 31396056

[B91] Spampinato S. F. Merlo S. Sano Y. Kanda T. Sortino M. A. (2017). Astrocytes contribute to Abeta-induced blood-brain barrier damage through activation of endothelial MMP9 . J. Neurochem. 142 (3), 464–477. 10.1111/jnc.14068 28488764

[B92] Spampinato S. F. Obermeier B. Cotleur A. Love A. Takeshita Y. Sano Y. (2015). Sphingosine 1 phosphate at the blood brain barrier: Can the Modulation of S1P receptor 1 influence the response of endothelial cells and astrocytes to inflammatory stimuli? PloS One 10 (7), e0133392. 10.1371/journal.pone.0133392 26197437 PMC4511229

[B93] Sultan S. Li L. Moss J. Petrelli F. Casse F. Gebara E. (2015). Synaptic integration of adult-born hippocampal neurons is locally controlled by astrocytes. Neuron 88 (5), 957–972. 10.1016/j.neuron.2015.10.037 26606999

[B94] Takeshita Y. Obermeier B. Cotleur A. C. Spampinato S. F. Shimizu F. Yamamoto E. (2017). Effects of neuromyelitis optica-IgG at the blood-brain barrier in vitro. Neurol. Neuroimmunol Neuroinflamm 4 (1), e311. 10.1212/NXI.0000000000000311 28018943 PMC5173350

[B95] Tanti A. Belzung C. (2013). Neurogenesis along the septo-temporal axis of the hippocampus: Are depression and the action of antidepressants region-specific? Neurosci. 252, 234–252. 10.1016/j.neuroscience.2013.08.017 23973415

[B96] Tao-Cheng J. H. Nagy Z. Brightman M. W. (1987). Tight junctions of brain endothelium in vitro are enhanced by astroglia. J. Neurosci. 7 (10), 3293–3299. 3668629 10.1523/JNEUROSCI.07-10-03293.1987 PMC6569185

[B97] Uberti D. Grilli M. Memo M. (2000). Contribution of NF-kappaB and p53 in the glutamate-induced apoptosis. Int. J. Dev. Neurosci. 18 (4-5), 447–454. 10.1016/s0736-5748(00)00018-6 10817929

[B98] Valente M. M. Allen M. Bortolotto V. Lim S. T. Conant K. Grilli M. (2015). The MMP-1/PAR-1 axis enhances proliferation and neuronal differentiation of adult hippocampal neural progenitor cells. Neural Plast 2015, 646595. 10.1155/2015/646595 26783471 PMC4691474

[B99] Valente M. M. Bortolotto V. Cuccurazzu B. Ubezio F. Meneghini V. Francese M. T. (2012). alpha2delta ligands act as positive modulators of adult hippocampal neurogenesis and prevent depression-like behavior induced by chronic restraint stress. Mol. Pharmacol. 82 (2), 271–280. 10.1124/mol.112.077636 22572885

[B100] Vallieres L. Campbell I. L. Gage F. H. Sawchenko P. E. (2002). Reduced hippocampal neurogenesis in adult transgenic mice with chronic astrocytic production of interleukin-6 . J. Neurosci. 22 (2), 486–492. 11784794 10.1523/JNEUROSCI.22-02-00486.2002 PMC6758670

[B101] Vogel D. Y. Kooij G. Heijnen P. D. Breur M. Peferoen L. A. van der Valk P. (2015). GM-CSF promotes migration of human monocytes across the blood brain barrier. Eur. J. Immunol. 45 (6), 1808–1819. 10.1002/eji.201444960 25756873

[B102] Wu L. Ye Z. Pan Y. Li X. Fu X. Zhang B. (2018). Vascular endothelial growth factor aggravates cerebral ischemia and reperfusion-induced blood-brain-barrier disruption through regulating LOC102640519/HOXC13/ZO-1 signaling. Exp. Cell Res. 369 (2), 275–283. 10.1016/j.yexcr.2018.05.029 29842876

[B103] Xia Y. P. He Q. W. Li Y. N. Chen S. C. Huang M. Wang Y. (2013). Recombinant human sonic hedgehog protein regulates the expression of ZO-1 and occludin by activating angiopoietin-1 in stroke damage. PloS One 8 (7), e68891. 10.1371/journal.pone.0068891 23894369 PMC3720889

[B104] Xing C. Wang X. Cheng C. Montaner J. Mandeville E. Leung W. (2014). Neuronal production of lipocalin-2 as a help-me signal for glial activation. Stroke 45 (7), 2085–2092. 10.1161/STROKEAHA.114.005733 24916903 PMC4122238

[B105] Yan M. Hu Y. Yao M. Bao S. Fang Y. (2017). GM-CSF ameliorates microvascular barrier integrity via pericyte-derived Ang-1 in wound healing. Wound Repair Regener. 25 (6), 933–943. 10.1111/wrr.12608 29328541

[B106] Yao Y. Tsirka S. E. (2014). Monocyte chemoattractant protein-1 and the blood-brain barrier. Cell Mol. Life Sci. 71 (4), 683–697. 10.1007/s00018-013-1459-1 24051980 PMC3946874

[B107] Yun S. Donovan M. H. Ross M. N. Richardson D. R. Reister R. Farnbauch L. A. (2016). Stress-induced anxiety- and depressive-like phenotype associated with transient reduction in neurogenesis in adult nestin-CreERT2/diphtheria toxin fragment A transgenic mice. PloS One 11 (1), e0147256. 10.1371/journal.pone.0147256 26795203 PMC4721672

[B108] Zand L. Ryu J. K. McLarnon J. G. (2005). Induction of angiogenesis in the beta-amyloid peptide-injected rat hippocampus. Neuroreport 16 (2), 129–132. 10.1097/00001756-200502080-00011 15671861

[B109] Zhang H. Zhang S. Zhang J. Liu D. Wei J. Fang W. (2018). ZO-1 expression is suppressed by GM-CSF via miR-96/ERG in brain microvascular endothelial cells. J. Cereb. Blood Flow Metab. 38 (5), 809–822. 10.1177/0271678X17702668 28430012 PMC5987931

[B110] Zhao F. Deng J. Yu X. Li D. Shi H. Zhao Y. (2015). Protective effects of vascular endothelial growth factor in cultured brain endothelial cells against hypoglycemia. Metab. Brain Dis. 30 (4), 999–1007. 10.1007/s11011-015-9659-z 25761767 PMC4491374

